# Assessing LGBTQ+ Legitimization in State Plans on Aging: The LGBTQ State Plan on Aging Report Card (LGBTQ-SPARC)

**DOI:** 10.1093/geroni/igaf122.005

**Published:** 2025-12-31

**Authors:** Megan McCoy

**Affiliations:** Northern Arizona University, Flagstaff, Arizona, United States

## Abstract

The 2020 reauthorization of the Older Americans Act (OAA) coupled with a federal rulemaking process effective in March 2024 together represent a historic shift in federal aging policy that acknowledges the importance of cultivating an aging services delivery system inclusive of LGBTQ+ older adults. Yet, to date there is no publicly available analysis of the extent to which states include LGBTQ+ older adults in the planning processes that determine how aging services funded under the OAA are implemented locally. Through a critical discourse analysis lens, we conducted a document review of current state plans on aging for all 50 states and the District of Columbia (N = 51) to assess the extent to which LGBTQ+ older adults are included in the language of state plans on aging and the context of that language in relation to key domains including: definitions of “greatest social need,” community engagement, data collection, and training/professional development. Findings indicate that while plans for 36 states contain LGBTQ+ inclusive terms, the context of that language and the extent to which it directly legitimizes LGBTQ+ older adults in key domains varies geographically. In addition to sharing findings with the research community, dissemination activities include creation of a publicly accessible LGBTQ State Plan on Aging Report Card (LGBTQ-SPARC) depicting level of LGBTQ+ inclusion by state, providing a snapshot of how states currently conform to best practices for LGBTQ+ inclusion in planning and serving as a baseline against which to measure LGBTQ+ inclusion in state planning processes for the future.

